# A personalized intervention to prevent depression in primary care based on risk predictive algorithms and decision support systems: protocol of the e-predictD study

**DOI:** 10.3389/fpsyt.2023.1163800

**Published:** 2023-06-02

**Authors:** Juan A. Bellón, Alberto Rodríguez-Morejón, Sonia Conejo-Cerón, Henar Campos-Paíno, Antonina Rodríguez-Bayón, María I. Ballesta-Rodríguez, Emiliano Rodríguez-Sánchez, Juan M. Mendive, Yolanda López del Hoyo, Juan D. Luna, Olaya Tamayo-Morales, Patricia Moreno-Peral

**Affiliations:** ^1^Biomedical Research Institute of Malaga (IBIMA Plataforma Bionand), Málaga, Spain; ^2^Prevention and Health Promotion Research Network (redIAPP), ISCIII, Madrid, Spain; ^3^Network for Research on Chronicity, Primary Care, and Prevention and Health Promotion (RICAPPS), ISCIII, Madrid, Spain; ^4^‘El Palo' Health Centre, Servicio Andaluz de Salud (SAS), Málaga, Spain; ^5^Department of Public Health and Psychiatry, University of Málaga (UMA), Málaga, Spain; ^6^Department of Personality, Evaluation and Psychological Treatment, University of Málaga (UMA), Málaga, Spain; ^7^Centro de Salud San José, Distrito Sanitario Jaén Norte, Servicio Andaluz de Salud (SAS), Linares, Jaén, Spain; ^8^Centro de Salud Federico del Castillo, Distrito Sanitario Jaén, Servicio Andaluz de Salud (SAS), Jaén, Spain; ^9^Unidad de Investigación de Atención Primaria de Salamanca (APISAL), Gerencia de Atención Primaria de Salamanca, Instituto de Investigación Biomédica de Salamanca (IBSAL), Salamanca, Spain; ^10^Department of Medicine, University of Salamanca (USAL), Salamanca, Spain; ^11^‘La Mina' Health Centre, Institut Català de la Salut (ICS), Barcelona, Spain; ^12^Instituto de Investigación Sanitaria de Aragón (IISA), Universidad de Zaragoza (UNIZAR), Zaragoza, Spain; ^13^Department of Statistics and Operational Research, University of Granada (UGR), Granada, Spain

**Keywords:** depression, prevention, internet-based interventions, mobile applications, primary health care, randomized controlled trial

## Abstract

The predictD is an intervention implemented by general practitioners (GPs) to prevent depression, which reduced the incidence of depression-anxiety and was cost-effective. The e-predictD study aims to design, develop, and evaluate an evolved predictD intervention to prevent the onset of major depression in primary care based on Information and Communication Technologies, predictive risk algorithms, decision support systems (DSSs), and personalized prevention plans (PPPs). A multicenter cluster randomized trial with GPs randomly assigned to the e-predictD intervention + care-as-usual (CAU) group or the active-control + CAU group and 1-year follow-up is being conducted. The required sample size is 720 non-depressed patients (aged 18–55 years), with moderate-to-high depression risk, under the care of 72 GPs in six Spanish cities. The GPs assigned to the e-predictD-intervention group receive brief training, and those assigned to the control group do not. Recruited patients of the GPs allocated to the e-predictD group download the e-predictD app, which incorporates validated risk algorithms to predict depression, monitoring systems, and DSSs. Integrating all inputs, the DSS automatically proposes to the patients a PPP for depression based on eight intervention modules: physical exercise, social relationships, improving sleep, problem-solving, communication skills, decision-making, assertiveness, and working with thoughts. This PPP is discussed in a 15-min semi-structured GP-patient interview. Patients then choose one or more of the intervention modules proposed by the DSS to be self-implemented over the next 3 months. This process will be reformulated at 3, 6, and 9 months but without the GP–patient interview. Recruited patients of the GPs allocated to the control-group+CAU download another version of the e-predictD app, but the only intervention that they receive via the app is weekly brief psychoeducational messages (active-control group). The primary outcome is the cumulative incidence of major depression measured by the Composite International Diagnostic Interview at 6 and 12 months. Other outcomes include depressive symptoms (PHQ-9) and anxiety symptoms (GAD-7), depression risk (predictD risk algorithm), mental and physical quality of life (SF-12), and acceptability and satisfaction (‘e-Health Impact' questionnaire) with the intervention. Patients are evaluated at baseline and 3, 6, 9, and 12 months. An economic evaluation will also be performed (cost-effectiveness and cost-utility analysis) from two perspectives, societal and health systems.

**Trial registration:**

ClinicalTrials.gov, identifier: NCT03990792.

## Introduction

According to the World Health Organization (WHO) reports, 322 million people worldwide are affected by depression (1). In addition, during the COVID-19 pandemic, the prevalence of depressive disorders increased by 27.6% (2). Depressive disorders have a high probability of recurrence and, in some cases, a tendency to become chronic. Only 32% of depressive disorders remained consistently recovered at 9 years of follow-up (3). Depression is associated with other mental (4) and physical illnesses (5, 6) and generates a high mortality rate among people who suffer from it (7). Depression ranked second in the number of years lived with disability (YLDs) (8). Depression also has significant economic consequences due to the increased use of health services, pharmacological and psychological treatments, and, to a greater extent, due to sick leave and loss of labor productivity (9). The 12-month prevalence of major depression reaches 11% of general practice attendees across Europe (10) and more than 70% of people suffering from depression consulted their GPs for this reason (11). However, “*the declining halves rule*” (12) is a reality: <50% of primary care attendees with depressive disorders were correctly diagnosed (13). Of these, <50% received adequate treatment (pharmacological or psychological) (14), and of these, <50% were adherent (15).

Even in the hypothetical situation where all cases of depression were correctly diagnosed and treated and patients were adherent, the disease burden of depression (YLDs) could only be reduced by 30% (16). This limitation occurs, among other reasons, because current treatments for depression are not as effective as we would like (17) and because of the so-called preventive gap. Approximately 40% of the 12-month prevalence of depression is due to the continuous occurrence of new cases of depression (10, 18). Therefore, an additional way to reduce the burden of depression is to lower the incidence of new cases, which can be achieved through prevention rather than treatment (19). We will use the term “interventions” to prevent depression only for those that were implemented before the onset of a depressive disorder (20). In the last two decades, dozens of systematic reviews and meta-analyses on psychological and psycho-educational interventions to prevent depression have been conducted (21). Nowadays, there is an agreement that psychological and psycho-educational interventions are effective, although their preventive effect is small, between 20 and 40% reduction in the incidence of depression compared to the control group (21, 22). From a public health point of view, this small effect size in relative terms could be clinically relevant in absolute terms (avoided depression, improvements in quality of life, and cost reduction) if preventive interventions were cost-effective (23) and scalable to a large number of people at risk. This could be achieved in the workplace (24), school settings (25), and primary care (26), and in a more transversal way, through information and communication technologies (ICTs) (27).

The predictD-Spain is a risk algorithm to predict the onset of episodes of major depression at 12 months in primary care (28). Free access to web calculators based on predictD algorithms is available at http://www.predictplusprevent.com/index.php?idioma=en. Based on the predictD risk algorithm, we developed the predictD intervention, which was evaluated in a cluster randomized trial with 3,326 non-depressed primary care attendees (29). The predictD intervention is a personalized biopsychosocial intervention implemented by GPs with five components: (1) a training workshop for GPs; (2) GPs communicating the level and risk profile of depression to patients in a 15-min interview every 6 months; (3) developing a personalized biopsychosocial intervention to prevent depression in these GP-patient interviews; (4) offering a psychoeducational booklet; and (5) activating and empowering patients (30). The predictD intervention vs. usual care reduced the incidence of major depression over 18 months by 21% (29) and another 21% for anxiety (31). The predictD intervention was cost-effective compared with usual care (32). There was good acceptance and adherence by patients and GPs (90% of patients and GPs carried out at least two of the three protocol visits), and no side effects were found (29). Patients were pleased to be informed about their risk for depression (33). GPs were comfortable and satisfied with the predictD intervention, suggesting some areas for improvement. GPs also identified some barriers such as workload, lack of time, and low motivation to address mental health problems in primary care (34).

Internet- and mobile-based mental health interventions have garnered increasing attention in the last decade (35). Among other reasons, more than three billion people had their own smartphone in 2021. Other reasons for this interest lie in the advantages they offer compared to traditional interventions: (1) users remain anonymous and thus have more privacy, avoiding stigmatization; (2) individuals can choose when, where, and their own pace of work; (3) lower economic costs; (4) ease of access to a wider range of people (disabled population, rural areas, etc.); and (5) reduction in waiting time. However, this type of intervention also has limitations and concerns (36). Although there are over 10,000 commercially available mobile-based mental health applications (apps), very few are based on randomized clinical trials that support their effectiveness, and there are few resources available to help end users (patients, clinicians, and healthcare organizations) to evaluate the quality and suitability of these products. Many patients and end users stop using an app 2 weeks after download, and low adherence to the intervention is the most common issue (36). In addition, these interventions are less accessible to elderly individuals and those with low digital expertise. Finally, this type of intervention also raises privacy and ethical concerns. We recently conducted a meta-analysis on the effectiveness of internet- and mobile-based mental health interventions to prevent depression (27). We found a small preventive effect and the quality of evidence was moderate. Therefore, given that these interventions are very accessible and can be applied on a wide scale, they should be further developed, evaluated, and implemented.

We propose four innovative approaches for the prevention of depression: (1) until now, the vast majority of interventions to prevent depression have had a psychological orientation. From the predictD intervention, we proposed a broader orientation, “the biopsychosocial orientation” (29, 30). Since there are different types of evidence-based preventive interventions for depression [e.g., psychological (21, 22), exercise-based (37), or social-support based (38)] with comparable effect sizes, these could be offered to people, alone or in combination, taking into account their risk level and specific modifiable risk factors (e.g., cognitive distortions, sedentary lifestyle, and/or low social support). (2) The key idea of personalized medicine is to base medical decisions on individual patient characteristics rather than on population averages (39). Frequently, personalized medicine is related to the identification of genetic and molecular factors for the application of individualized treatments (*precision medicine*), such as in the case of breast cancer treatment. In psychiatry, this precision medicine approach is beginning to be investigated for the choice of pharmacological treatments (40). Moreover, predicting optimal outcomes (*predictive medicine*) has also been used to try to personalize psychological treatments (41). Numerous investigations of predictors or moderators of treatment response in psychiatric disorders have been reported over the past decades, including clinical features as well as biological measures, and also recently for the prevention of depression (42, 43). However, none of these have entered routine clinical practice (44). In primary care, there is a high degree of *personalized prevention* of cardiovascular diseases integrated into routine clinical practice. Therefore, the concept and strategy of developing “personalized prevention plans” is not new. The real innovation is to apply personalized prevention plans (PPPs) to the prevention of depression. Until now, psychological intervention programs to prevent depression and clinical trials designed to test their effectiveness relied on all patients performing the same intervention. What we propose in the e-predictD study is that each user implements only the components of the intervention that are truly indicated according to his/her risk level (quantitative risk) and risk profile (qualitative risk) of depression. (3) We were also pioneers in the use by GPs of “validated risk algorithms to predict depression” to personalize the prevention of depression (29, 30). We will use these once again in this study, but here, the information on the level and profile of risk of depression will be obtained directly by patients from an app installed on their smartphones. (4) Creating personalized plans to prevent depression and effectively implementing them is in itself an innovative challenge. Our proposal is based on technological solutions: automatic integration of multiple inputs (user's information) through prediction and decision algorithms by “decision support systems (DSSs),” the outputs of which meet a basic rule: “all information given to the end user must be accompanied by a brief and reasoned suggestion concerning what to do, which will be based on scientific evidence when available.” Finally, it is necessary to integrate a “monitoring and feedback system” that, on the one hand, generates new inputs for the DSS and, on the other hand, keeps the user's motivation high without overwhelming him/her with an excess of information and control. Computerized clinical DSSs are information technology systems that deliver patient-specific recommendations to clinicians to promote improved care (45). These DSSs may be integrated into provider electronic health records, accessed through the Internet, or delivered through mobile devices. Most DSSs focused on depression have been developed to improve the quality of clinical decisions (46). There is limited experience in DSSs for patients and even less in DSSs coordinated between patients and health professionals. A similar DSS to the e-predictD DSS, which includes the use of predictive algorithms, has recently been evaluated regarding its feasibility for the treatment of depression (47). However, to our knowledge, none have been developed and tested for the prevention of depression thus far.

### Objectives

The overall aim of the e-predictD study is to design, develop, and evaluate a personalized intervention for the prevention of depression based on ICTs, risk prediction algorithms, DSSs, and PPPs. The specific objectives are as follows:

1. To design and develop a new personalized intervention to prevent depression, the e-predictD intervention, from an evolved predictD intervention based on ICTs, risk predictive algorithms, DSSs, and PPPs.
2. To evaluate the effectiveness of the e-predictD intervention in preventing the onset of major depressive episodes compared to an active control group.
3. To evaluate the effectiveness of the e-predictD intervention in reducing depression and anxiety symptoms and depression risk compared to an active control group.
4. To evaluate the effectiveness of the e-predictD intervention in increasing the mental and physical quality of life compared to an active control group.
5. To evaluate the patients' acceptability and satisfaction with the e-predictD intervention compared to an active control group.
6. To evaluate the cost-effectiveness and cost-utility of the e-predictD intervention in preventing depression compared to an active control group.

## Methods and analysis

### Design

A parallel two-arm randomized controlled trial with cluster assignment by GP, allocation ratio 1:1, and simple blind was conducted. This clinical trial compared the e-predictD plus care-as-usual (+CAU) intervention to prevent depression with an active + CAU control group. Primary and secondary outcomes were assessed for 12 months. Assessments took place at baseline and 3, 6, 9, and 12 months of follow-up. This protocol is reported according to the standard protocol items for clinical trials (*SPIRIT statement*) (48, 49) and taking into account the consolidated standards of reporting trials *CONSORT for cluster randomized trials* (50) as well as the guidelines for executing and reporting internet (*CONSORT e-Health*) (51) and social and psychological interventions (***CONSORT-SPI***) (52). This study was prospectively registered on ClinicalTrials.gov: NCT03990792. The current trial status is: active, not recruiting.

### Selection of subjects

#### General practitioners and primary care centers

We planned to recruit 72 GPs (see below “power and sample size calculation” section) who did not intend to change their workplace in the next 12 months. GPs were informed of the study through a meeting that the research team held in those primary care centers (PCCs) that wished to participate. GPs who signed the informed consent form to participate in the study were informed that later they would be randomly, centrally, and blindly assigned to the intervention or active control group through a computer program. Participating GPs were employed at PCCs in six Spanish cities: Malaga, Jaen, and Linares in the south, Salamanca in the center-west, Zaragoza in the north, and Barcelona in the north-east of Spain. PCCs in Spain cover a population of 15,000 to 30,000 inhabitants from a geographically defined area. GPs in each PCC work with other primary care professionals (nurses, pediatricians, social workers, and administrative staff) as a group. The Spanish National Health Service provides free medical cover to over 99% of the population. Patients can visit their GPs as often as they wish free of charge, even when they do so for preventive reasons. Each patient is assigned to only one GP, who has gatekeeper functions.

#### Patients

Patients were recruited in PCCs from the list of patients assigned to each GP. The recruitment strategy consisted of GPs checking all patients from their appointment lists to determine if they met any of the following exclusion criteria based on the data contained in their primary care electronic health records: age under 18 or over 55 years; inability to understand or speak Spanish; severe mental disorder (psychosis, bipolar, personality disorder…); cognitive impairment; and/or terminal illness. GPs briefly presented the e-predictD study to all those patients who did not meet any of the exclusion criteria. If patients were interested in the study, the GP referred them to contact a research assistant placed in an adjoining room. Patients who contacted the research assistants received a more extensive explanation of the study and had the opportunity to discuss and ask questions about it. The research assistants gave them an informative brochure about the e-predictD study and the consent form to be signed if they wanted to participate in the screening. The research assistants verified whether the participants who signed the informed consent form met the following inclusion criteria: (1) have a Smartphone, tablet, or computer with an internet connection for the next 12 months; (2) will remain in their place of residence in their respective cities for at least the next 9 months; (3) obtain a score ≤10 on the Patient Health Questionnaire (PHQ-9) questionnaire (53); and (4) have a probability of depression in the next year ≥10% (predictD risk algorithm) (38). The research assistants used a web application to record the answers of the participants and these were automatically incorporated into the database along with the rest of the self-completed measures at baseline. For a prevalence of depression in primary care attendees between 10%−15% and a PHQ-9 score ≤10, the negative predictive value is over 94% (≤5% false negatives) (54); therefore, we were very sure that we included non-depressed patients in our study. Using 10% as the probability threshold for depression next year, we guaranteed that patients included in our study had a moderate or high risk of depression. Exclusion and inclusion criteria are summarized in Box 1.

Box 1 **Exclusion and inclusion criteria in the e-predictD study.**

Exclusion criteria

GPs will be excluded if they…

- …are planning to change their place of work in the next 12 months.

Patients will be excluded if they…

- …are aged under 18 or over 55 years.
- …are unable to understand or speak Spanish.
- …have a documented severe mental disorder (psychosis, bipolar disorder, personality disorder…), cognitive impairment, or terminal illness.

Inclusion criteria

Patients will be included if they…

- …have a smartphone, tablet, or computer with an internet connection.
- … will maintain their place of residence in the respective cities for at least 9 months of the next year.
- …Obtain a baseline PHQ-9 score <10.
- …have a baseline probability of the onset of major depression at 12 months ≥10% according to the predictD risk algorithm.

Patients who had no exclusion criteria and met all inclusion criteria were informed in writing by the research assistants that two interventions (interventions A and B) would be tested to prevent depression, they would be randomly assigned to one of them, and that both interventions will be implemented online, through their smartphones or/and tablet-computers. Once the patient information and selection process was complete, the research assistants gave them the second consent form to be signed if they wanted to participate in the trial. If this last informed consent is signed, then the research assistants provided participants with the web address of the e-predictD app and an access code to download the app. In addition, the research assistants were available to help patients install the app on their smartphones if necessary.

**Figure 1:**
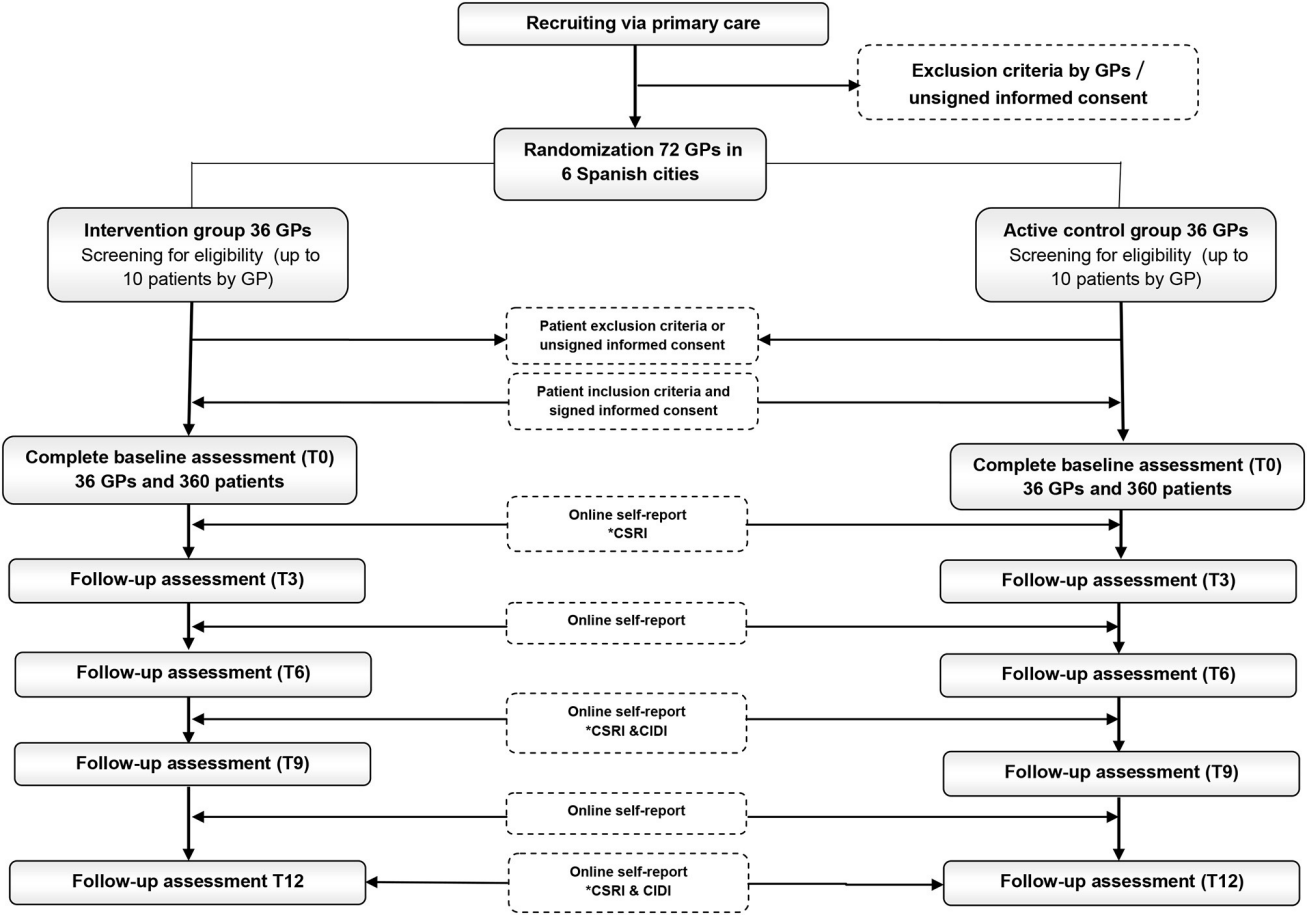
Recruitment and follow-up assessments of GPs and primary care attendees. *Hetero-administered questionnaires for patients: CSRI, Client Service Receipt Inventory; CIDI, Composite International Diagnostic Interview.

We anticipated recruiting a total of 720 patients (10 for each of the 72 GPs) of which about 360, under the care of 36 GPs would be assigned to each of the two conditions (see below the “power and sample size calculation” section). Recruitment was affected by the COVID pandemic and only 69 GPs and 633 patients were recruited. The entire selection process for GPs and patients and the different evaluations over the 12-month follow-up are summarized in Figure 1.

### Randomized allocation and masking

We conducted a centralized, blinded, and randomized allocation of GPs to the e-predictD intervention + CAU or the active control + CAU group. An independent person from outside the research team performed the allocation through a computer program. This randomized allocation process was stratified by city. All primary care attendees recruited were automatically allocated to the same group as their GPs to avoid contamination bias between patients. To minimize selection bias attributable to GPs in the patient recruitment process, GPs were required to offer participation in the e-predictD study to all primary care attendees who met the selection criteria. During each day of recruitment, the research assistants checked that the latter was fulfilled.

The GPs were not blinded to their assignment to the intervention or active control group; which is common when health professionals implement psychosocial interventions in clinical trials conducted in the real world (55). However, primary care attendees were blinded to their assignment allocation, because they were informed that two interventions (A and B) would be tested to prevent depression and they would be randomly assigned to one of them. In addition, both intervention and active control groups received the same usual care and some type of intervention to prevent depression (see “interventional methods” section), filled in exactly the same self- and hetero-administered questionnaires, and the respective versions of the app that were downloaded on their smartphones had the same appearance. The research assistants who collaborated in the recruitment of GPs and patients and evaluated some outcomes through interviews (only for the Client Service Receipt Inventory (56) and the Composite International Diagnostic Interview, CIDI-depression) (57) were blinded to their allocations. Those who will perform statistical analyses will also be blinded to the intervention and active-control codes in the databases.

### Interventional methods

The e-predictD intervention is a complex intervention that has multiple active ingredients interacting with each other, enhancing its final preventive effect. The UK Medical Research Council proposes a framework of four phases for developing and evaluating complex interventions: development, feasibility/piloting, evaluation, and implementation (58, 59). In the e-predictD study, we are carrying out the first three phases. Below, we describe their first four steps.

1. *Reviewing published evidence*: In recent years, our research team, through systematic reviews and meta-analyses, has been evaluating different potentially effective interventions in the prevention of depression such as physical exercise-based (37) and social-based (38) nterventions and also those for which more evaluations are available, psychological and psychoeducational interventions (21, 22), as well as evaluations of the latter in specific implementation contexts, in primary care (26) and via the Internet- and mobile-based interventions (27). From all these meta-analyses, it can be concluded that such interventions have a preventive effect on depression, although their effect size is small. In addition, it is important to note that the e-predictD intervention is based on the predictD intervention (29, 30), a personalized biopsychosocial intervention implemented by GPs in primary care that included a predictive risk algorithm for depression, which reduced the incidence of depression-anxiety (29, 31) and was cost-effective (32).
2. *Drawing on existing theories*: There are four possible mediators through which depression can be prevented (60): (a) behavioral activation: reinforcing or initiating behaviors for which there is scientific evidence of their involvement in the prevention of depression, (b) cognitive changes: changing beliefs and/or ways of managing mental activity, (c) learning to regulate negative emotions and fostering positive emotions; and (d) physiological changes. Any change in one of these mediators could trigger changes in the others. For example, if a person is sedentary and decides to do more physical exercise (behavioral change) (37), he/she will feel more relaxed (emotional change) through physiological changes [increased beta-endorphins, optimization of monoamines, tryptophan, and endocannabinoids systems, changes in BDNF activity, reduction of inflammatory processes (oxidative stress and cytokines), and increased hippocampal cell proliferation] (61) and that may help him/her to be more positive (cognitive change).

It is also understood that behavior, cognition, emotion, and physiology can respond both to intrapersonal factors (e.g., when a person decides to do something different, feels very frustrated when he/she does not do things well, or is paralyzed in dangerous situations) and interpersonal triggers (e.g., when a person works better when his/her effort is recognized, feels that his/her father's passivity infuriates him/her, or considers that being with his/her daughter calms him/her down). In addition, we build beliefs in interaction with other people, so a large part of our beliefs are the product of exchanges with other people. It has also been postulated that social support has a direct (its lack is a stressor itself) and/or indirect (mitigating the effect of other stressors) effect on depression prevention (38).

**Figure 2:**
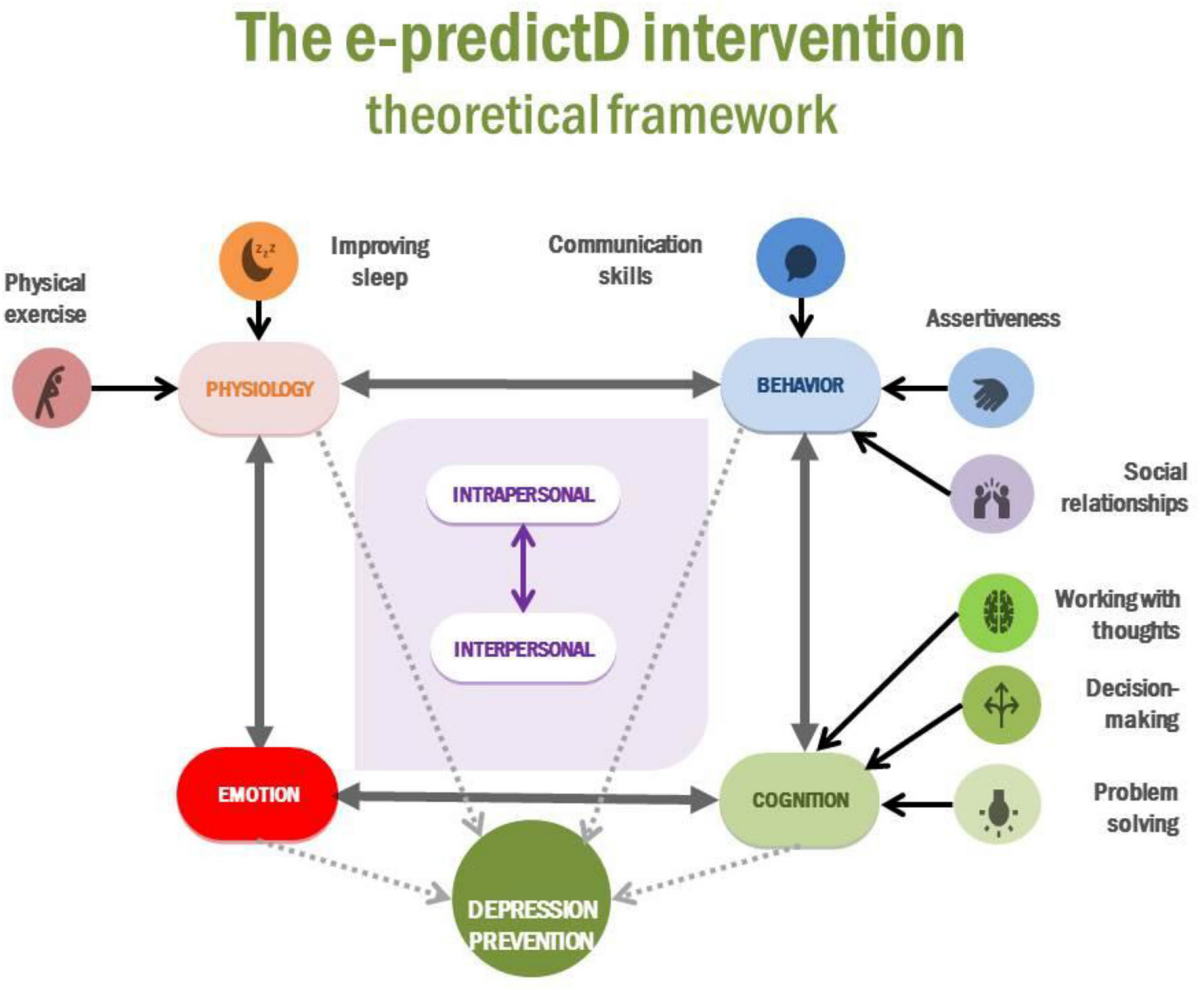
Theoretical framework of the e-predictD intervention.

The objective of the e-predictD intervention is to offer a personalized combination of eight intervention modules (see below “the e-predictD intervention modules” section) that allow an individual to initiate or/and maintain positive changes in any of the four mediators to prevent depression. Physical exercise and improved sleep are aimed at improving physiological aspects. Communication skills, assertiveness, and social relationships promote behavioral changes to facilitate new interactions with other people. Problem-solving, decision-making, and working with thoughts are aimed at fostering cognitive changes. The theoretical framework of the e-predictD intervention is represented by a logic model in Figure 2.

3) *Working with stakeholders and iterative cycles of developing versions of the intervention:* A multi-professional team made up of GPs, psychologists, psychiatrists, specialists in public health and preventive medicine, statisticians, social workers, computer and telecommunications engineers, and primary care attendees worked together on the design, development, and evaluation of the different β versions of the e-predictD intervention until the final version 1.0 was obtained. All of them also participated in decision-making about the content, format, and delivery of the intervention.
4) *A pilot study* was conducted to evaluate an early β version of the e-predictD intervention on feasibility, acceptability, satisfaction, and potential harms, to estimate parameters useful in calculating the final sample size needed and to measure changes in secondary outcomes (depression risk, depressive and anxious symptoms, and quality of life). To achieve these goals, qualitative and quantitative research was conducted. In addition, after the specific informed consents were signed by the GPs and patients, video or audio recordings of GP–patient interviews were made to assess the fidelity of the GPs to the script of the semi-structured interview of the e-predictD intervention (see below the “GP training and the GP-patient interview” section) and to explore relationships between those elements that would define the quality of the interview (e.g., active listening, empathy, etc.) and the secondary outcomes. Six GPs (one for each Spanish city included in the study) and 56 primary care attendees participated in the pilot study, which had a follow-up of 3 months.

### The e-predictD intervention

**Figure 3:**
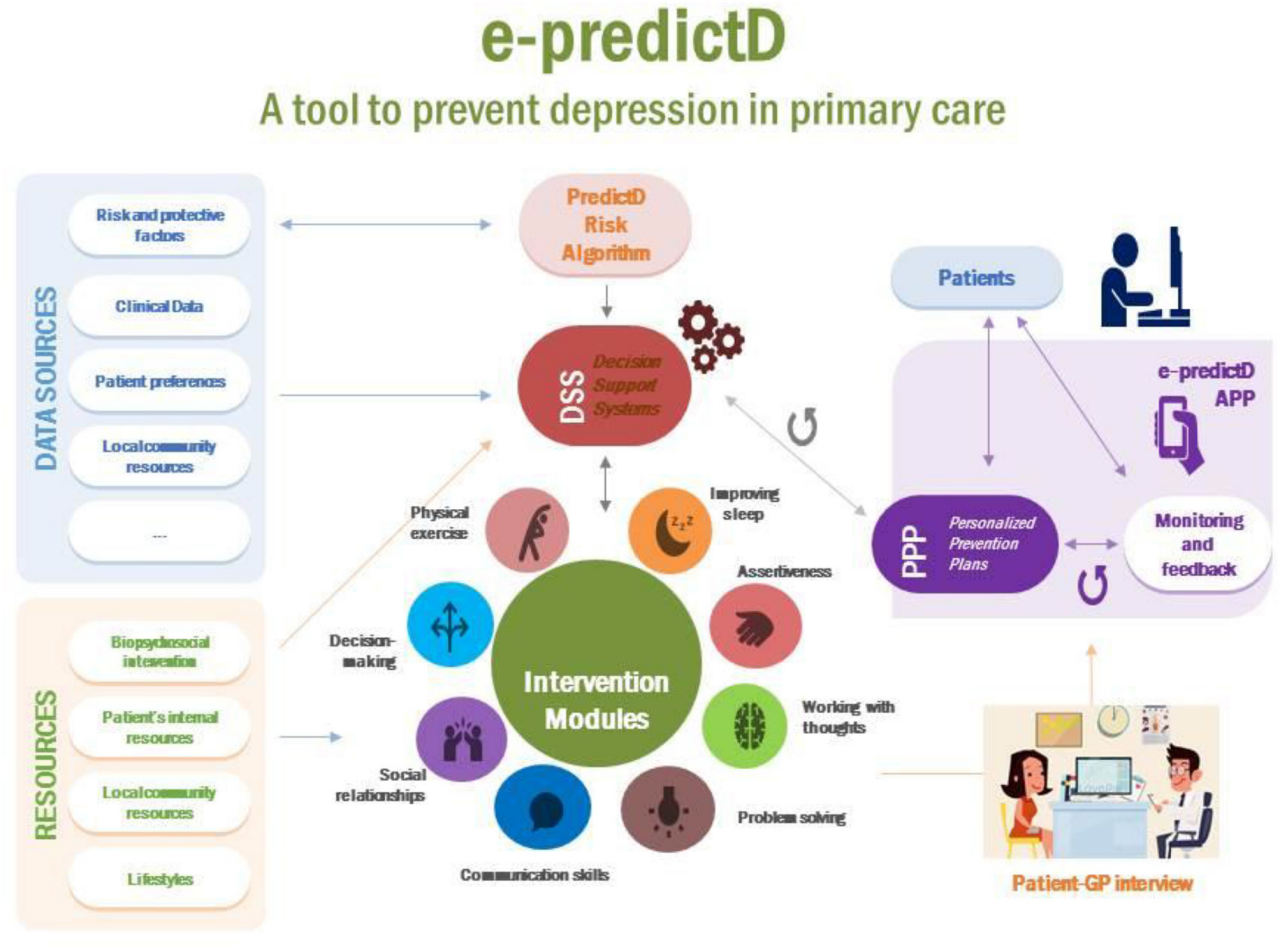
Operational diagram of the e-predictD intervention.

Participants allocated to the intervention arm will receive the “e-predictD intervention,” which is a biopsychosocial, multicomponent, and personalized intervention for the prevention of depression. An operational diagram of the e-predictD Intervention is described in Figure 3.

#### Personalized e-predictD reports for patients and GPs

**Figure 4:**
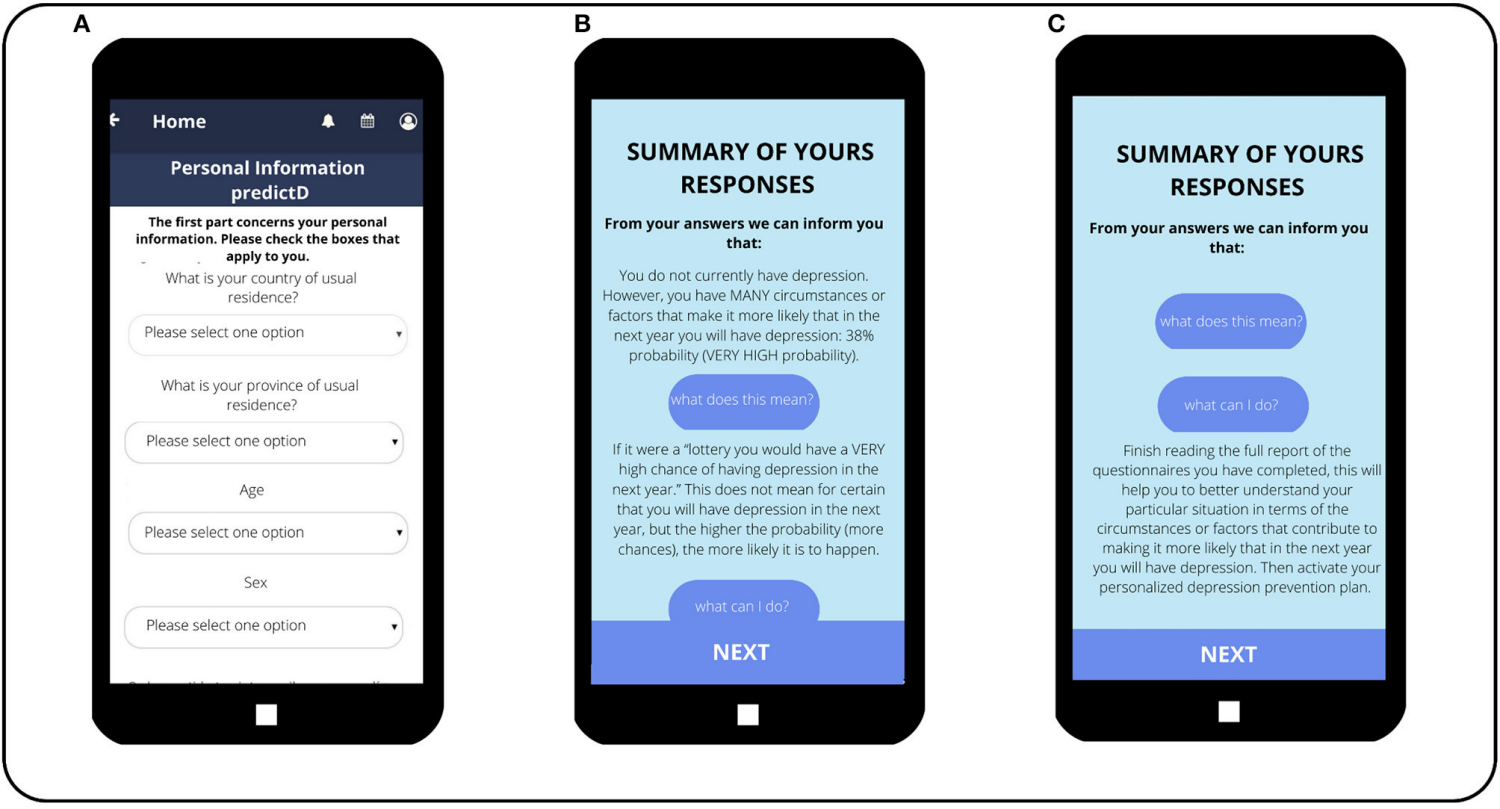
Self-filled questionnaire, e-predictD reports, and decision support system for patients (depression risk). **(Screen A)** The first questions of the questionnaire that patients complete on their own, once they have installed the e-predictD app on their phones. **(Screen B)** The report the patient receives upon completing the questionnaire. In this case, the patient is informed of his/her probability of depression risk. In addition, this patient clicked on the button “What does this mean?” so he/she is given a brief explanation of his/her probability of developing depression in the next year. **(Screen C)** The information the patient receives when he/she clicks on the button “What can I do?” regarding the information about having a 38% probability of depression.

**Figure 5:**
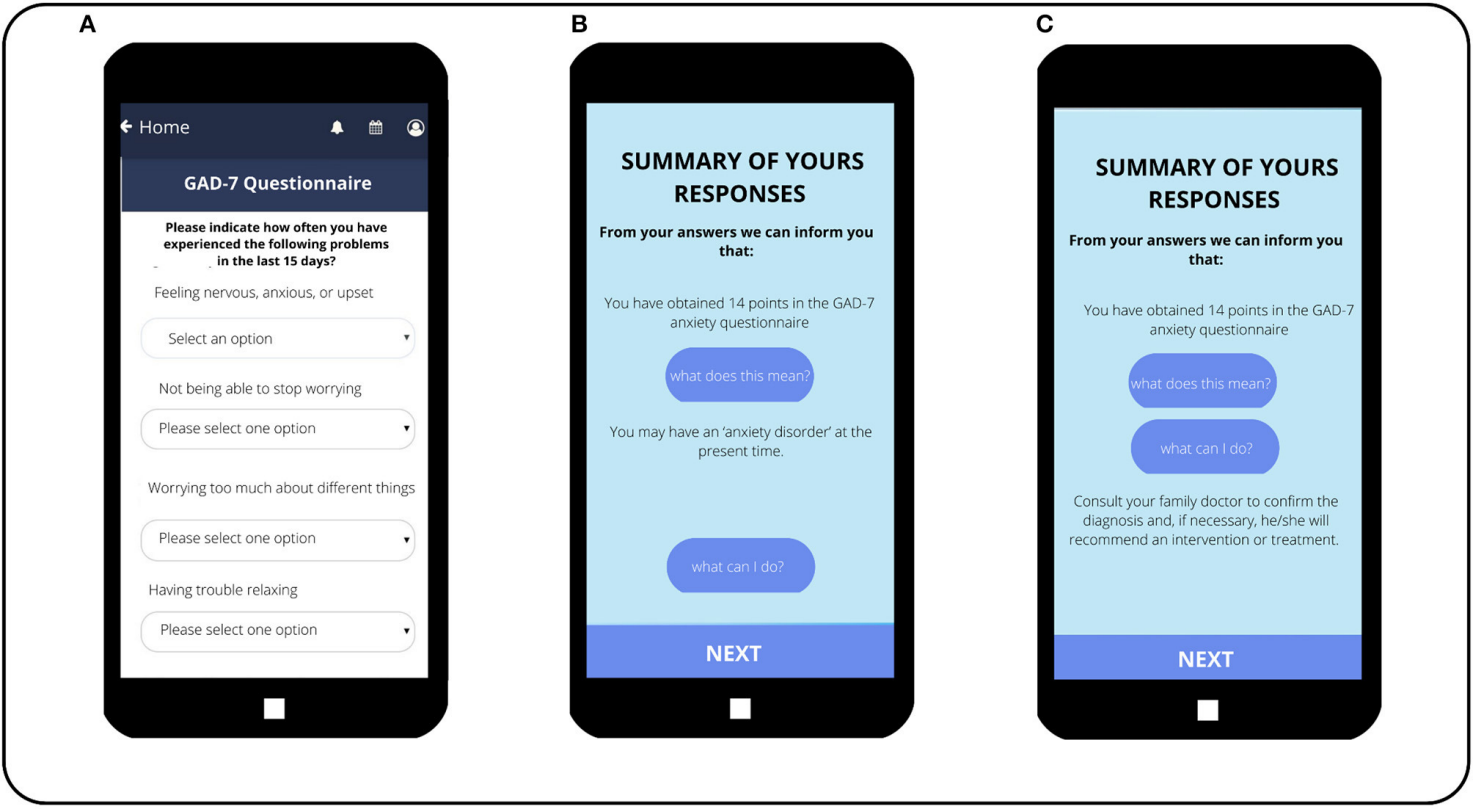
Self-filled questionnaire, e-predictD reports, and decision support system for patients (GAD-7 questionnaire). **(Screen A)** The first questions of the GAD-7 questionnaire that patients self-administer on their phones. **(Screen B)** The report the patients receive when they click on “What does this mean?” **(Screen C)** The report the patients receive when they click on “What can I do?”.

**Figure 6:**
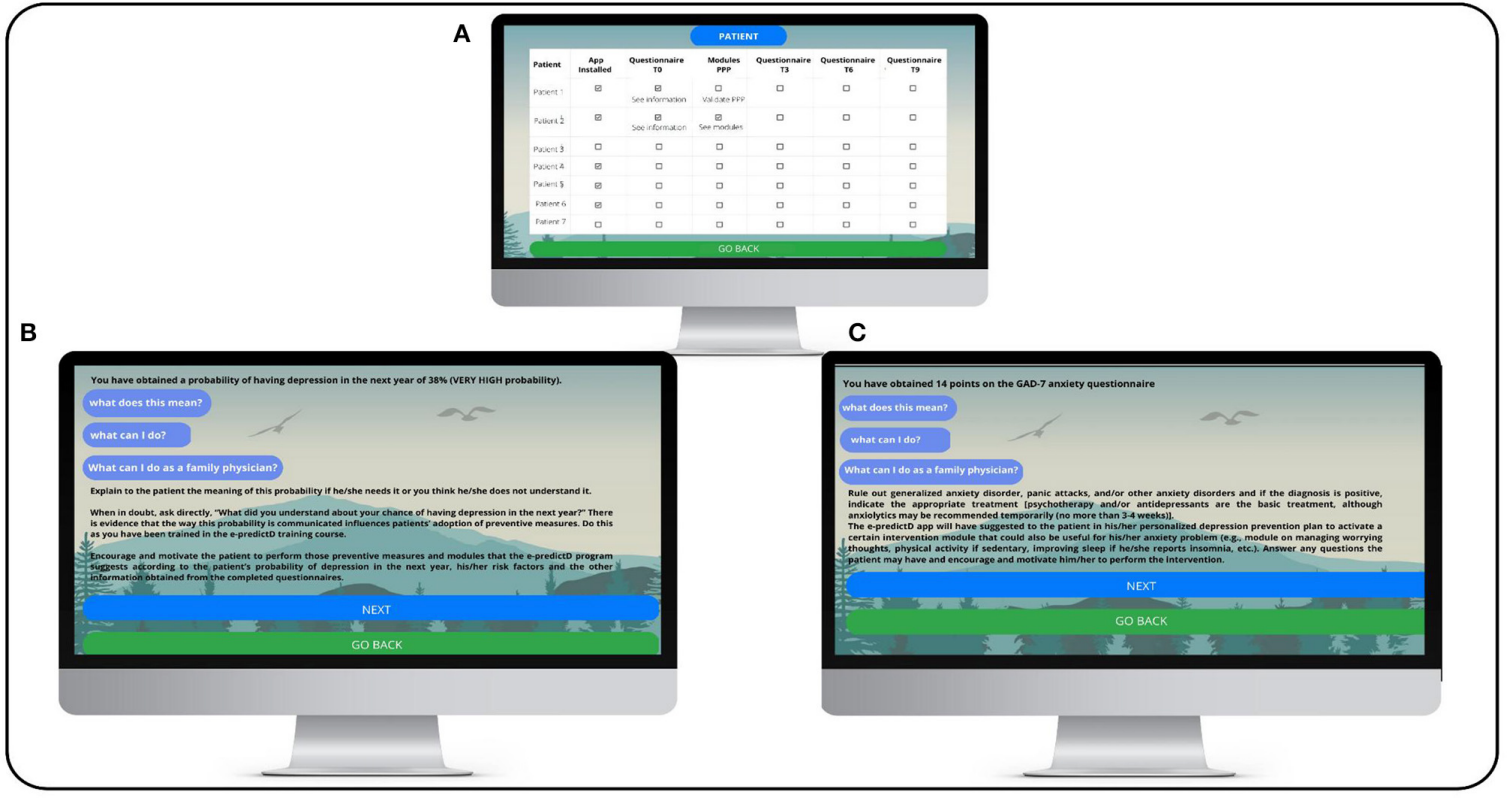
e-predictD reports and decision support system for GPs (risk depression and GAD-7). **(Screen A)** The GP can see the list of patients joining the e-predictD study on the computer in his/her primary care center office. Patient 1 has installed the app and completed the baseline questionnaires, but his/her personalized prevention plan (PPP) has not been activated. Patient 2, however, has activated the recommended intervention modules in his/her PPP. **(Screen B)** This screen shows the information that the GP sees when clicking on “View information” (Screen A) of the questionnaires that each patient has completed. Specifically, the result of the 38% probability of depression in the next year is displayed, as well as the “What does this mean?” and “What can I do?” buttons that also appear to the patient. In addition, exclusively for the GP, the button “What can I do as a GP?” also appears, which, if clicked, provides indications on how to inform and what to suggest to the patient. **(Screen C)** This screen shows the information that the GP sees when clicking on “View information” (Screen A) of the questionnaires that each patient has completed. Specifically, the result of 14 points on the GAD-7 questionnaire is displayed, as well as the “What does this mean?” and “What can I do?” buttons that also appear to the patient. In addition, exclusively for the GP, the button “What can I do as a GP?” also appears, which, if clicked, provides indications on how to inform and what to suggest to the patient.

Patients, who do not have exclusion criteria, meet all inclusion criteria and sign the informed consent which is provided with an access code so that they can download the e-predictD app from their App Store or Google Play. The e-predictD intervention is free for all participants in the e-predictD study and has a responsive design that can be used in different formats: smartphone, tablet, or/and computer (desktop or laptop). Patients can access the e-predictD intervention whenever they want through their username and password. The latter can be changed whenever they wish, and there is also a password recovery system if it is forgotten. Once the app is downloaded, patients are asked to self-complete a set of questions and questionnaires online (see Table 1). All the information generated from this set of questionnaires is automatically returned to each participant through a complete but brief personalized report, using non-technical language. For each section of this e-predictD report, participants can open a pop-up window about “What does this mean?” and “What can I do?” (See Figures 4, 5). A basic rule of the DSS is that “all information given to the end user, patients, and GPs must be accompanied by a brief and reasoned suggestion concerning what to do, which will be based on scientific evidence when available.” This e-predictD report is also sent to the patients' email in case they want to save, print, or consult it at another time. If the patients give their consent, their GPs will have access to their e-predictD reports from PCC desktop computers. GPs can also open pop-up windows about “What does this mean?” and “What can I do” (see Figure 6), but in this case, the information provided by the DDS is adapted to the GP's background and context. Therefore, the e-predictD DSS is designed to help decision-making for both patients and GPs.

**Table 1:** Overview of the assessments.

**Instruments**	**Aim**	**Data collection method**	**Timing (month)**					
			**Screening**	**T0**	**T3**	**T6**	**T9**	**T12**
Screening instruments								
PHQ-9	Depressive symptoms	Face to face	x					
predictD risk algorithm	Probability of the onset of major depression at 12 months	Face to face	x					
Primary outcome								
CIDI	Diagnosis of major depression	Telephone				x		x
Secondary outcomes								
PHQ-9	Depressive symptoms	Online self-report			x	x	x	x
predictD risk algorithm	Probability of the onset of major depression at 12 months	Online self-report			x	x	x	x
GAD-7	anxious symptoms	Online self-report		x	x	x	x	x
SF-12	Physical and Mental Quality of life	Online self-report		x	x	x	x	x
EuroQol-5D	Quality Adjusted Life Years	Telephone		x		x		x
CSRI	Healthcare resource use, sick leave and presenteeism	Telephone		x		x		x
Other assessments
Duke-UNC-2	Perceived functional social support	Online self-report		x	x	x	x	x
BPAA	Physical activity	Online self-report		x	x	x	x	x
Other questions	Socio-demographic variables	Online self-report		x	x	x	x	x
	Assertiveness	Online self-report		x	x	x	x	x
	Difficulties communicating with people	Online self-report		x	x	x	x	x
	Interpersonal problems	Online self-report		x	x	x	x	x
	Difficulties making a decision	Online self-report		x	x	x	x	x
e-HIQ-17	e-Health Impact Questionnaire	Telephone				x		x

PHQ-9, Patient Health Questionnaire-9; CIDI, Composite International Diagnostic Interview; GAD-7, Generalized Anxiety Disorder-7; SF-12, Quality of life-Short Form-12 items; BPAA, Brief Physical Activity Assessment for use by GPs; CSRI, Client Service Receipt Inventory.

#### Personalized plans to prevent depression

**Figure 7:**
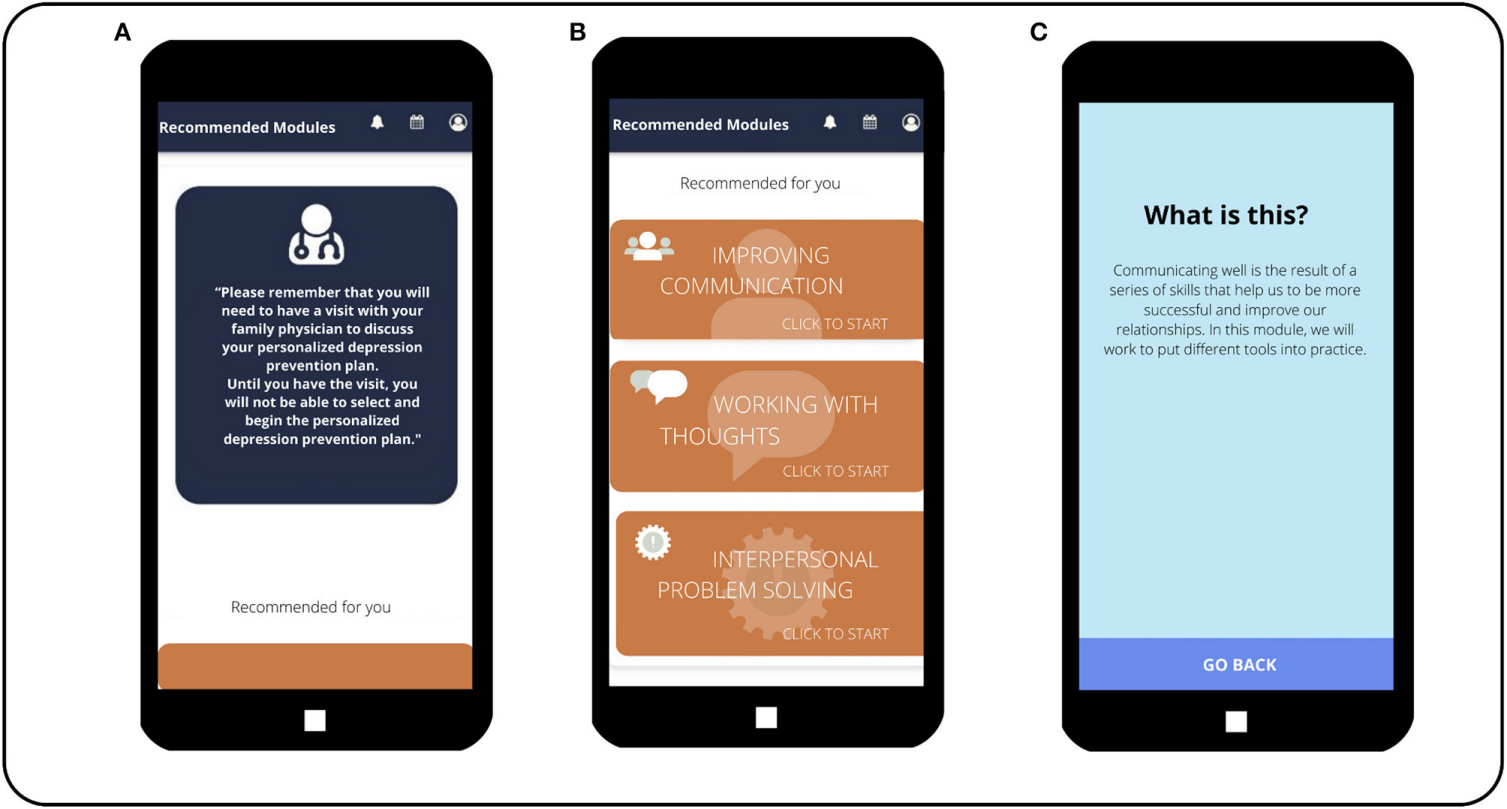
Personalized plans to prevent depression recommended to patients. **(Screen A)** Reminder message for the patient to make an appointment with his/her GP to discuss the personalized depression prevention plan recommended by the app. **(Screen B)**The three modules that have been recommended by the app for this patient are shown. Until the patient visits his/her GP and the GP confirms this, the patient will not be able to start working on the recommended modules and will only be able to access a brief description of them. **(Screen C)** Information that appears to the patient when he/she clicks on the “communication skills” module, before having a visit with his/her GP.

The DSS will help patients to choose their own PPP for depression integrating all the patients' information: risk and protective factors for depression, depression risk level (predictive risk algorithm “predictD”) (31), preferences, internal and external resources, clinical data, etc. Taking into account all these inputs and through pre-designed decision algorithms, the DSS will automatically offer each patient a PPP for depression, which will include different combinations of the eight intervention modules: physical exercise, social relationships, improving sleep, problem-solving, communication skills, decision-making, assertiveness, and working with thoughts (see the intervention modules section below). Patients will have the option to click on the pop-up sections (including brief and attractive summaries and some videos) to learn more about these intervention modules and how they can be useful to prevent depression. Some examples of PPP outputs are shown in Figure 7. Subsequently, the DSS releases at baseline the first personalized e-predictD report and its corresponding PPP, and then patients and their GPs are asked to arrange an appointment within the next 2 weeks to discuss their e-predictD reports and PPP. After the GP–patient interview (see the “GP training & GP-patient interview” section below), GPs must click on those intervention modules that, in their opinion, would be suitable for a given patient to use and could also suggest any other intervention module that was not chosen by the DSS algorithms. This option was considered under the assumption that the algorithms designed would have limitations that could somehow be overcome through the GP and the GP–patient interaction. It could happen that in the review of the patient's electronic health record by the GP or during the GP–patient interview, information could arise (clinical, emotional, family, or social) that was not detected by the DSS algorithms and the GP then thinks that another intervention module could be useful for a given patient. After the GP–patient interview, patients shall select at least one intervention module to be used for the next 3 months. However, if their proposed PPP includes more than one intervention module, among these, patients can use, simultaneously or consecutively, as many as they want. For the next 3 months, patients also have the option to stop using any of their chosen start-up intervention modules and begin using any of the other modules from their PPP. At 3, 6, and 9 months, patients will update the self-complete questionnaires and from these new inputs, the DSS will offer them a new personalized e-predictD report and PPP for the next 3 months; although in these cases, there is no specific GP–patient interview included in the e-predictD intervention protocol. Therefore, the e-predictD intervention's length will also be personalized to each participant. Some participants may choose a minimum intervention period, which involves a single intervention module for a few days or weeks. Meanwhile, others may need a more extended intervention, utilizing several modules throughout the year-long follow-up period.

#### GP training and the GP–patient interview

Although the e-predictD is basically a self-guided intervention to prevent depression delivered through an app, we decided to hold a GP–patient interview because it was the main active ingredient of the predictD intervention (29–32), giving the GP a facilitator role within the e-predictD intervention. Nonetheless, in this case, we reduced the number of GP–patient interviews to just one per year, as the GPs suggested in the qualitative evaluation of this intervention (34). Another active ingredient of the predictD intervention that we decided to maintain in the e-predictD intervention was specific training for GPs, since most GPs have had no training in depression prevention and none in the new e-predictD intervention.

The GP training consisted of 10 h of face-to-face group workshops during working hours and 5 h of individual online work solving real clinical cases taken from the pilot study. As this study was to be conducted in different Spanish cities, we carried out a training of the trainers to ensure that the GP training was conducted homogeneously and with a minimum quality standard followed in each of the cities. The 10 learning objectives of the GP training were to: (1) understand the e-predictD study in terms of its justification, design, intervention, follow-up, and security protocol; (2) know the risk and protective factors for depression and evidence-based interventions to prevent depression; (3) know how to interpret and communicate to each patient his/her level and profile of risk of depression; (4) undertake active listening about patients' beliefs, expectations, and the impact of information transmitted through e-predict reports and PPPs for depression; (5) know how to detect and empathetically manage patients' negative and positive emotions during the GP–patient interview; (6) know the eight intervention modules included in the e-predictD app; (7) know how to detect and cope with difficulties patients may have in understanding and following the e-predictD intervention; (8) know how to support those patients who are excessively worried by the information given; (9) know how to detect and manage the mental and physical comorbidities most frequently associated with patients at risk of depression; and (10) know how to prepare and conduct the single GP–patient interview of the e-predictD intervention. The methodology of the face-to-face group workshop was very interactive, including role-playing to learn skills, solving clinical cases, and analyzing video recordings of real GP–patient interviews carried out in the pilot study.

The GP–patient interview has two parts: the first addresses the preparation of the interview and the second is the interview itself. The first part begins when GPs receive notification that one of their patients has been included in the e-predictD study and his/her e-predictD report and respective PPP for depression are available. The GP will be able to access from his/her PCC desktop computer patient's information if consent was given. As stated previously, when the GPs are reading a patient's e-predictD report, they can open pop-up windows that answer two questions: “What does this mean?” and “What can I do?” Each reasoned response is organized and chosen by the DSS through predefined algorithms based on the availability of scientific evidence and adapted to the GP's context. In addition, the GPs are trained to search for biopsychosocial clinical data in their patient's electronic health records, which could be useful to help patients prevent depression. Among others, they will search physical and mental comorbidities (e.g., hypothyroidism, chronic pain, generalized anxiety disorder/panic attacks, and previous depressive episodes), family and social problems (e.g., loneliness, marital, or economic problems), and all types of treatments and interventions (e.g., antidepressants, anxiolytics, beta-blockers, and previous psychotherapies). Depending on the patient's complexity, 5–15 min should be devoted to this preparatory part of the GP–patient interview.

The only GP–patient interview included in the protocol of the e-predictD intervention has as its general objective to discuss the e-predictD report and its respective PPP for depression, build a therapeutic and patient-centered relationship, and help patients prevent depression. This is a semi-structured GP–patient interview whose script and associated objectives are described in Box 2. This GP–patient interview is designed to last an average of 15 min as a longer duration would not be feasible in a real practice setting in primary care (34). All the GPs in the intervention group received practical training to carry out this interview.

Box 2

**Table:** Semi-structured patient–GP interview to prevent depression.

**Objectives**	**Script examples**
1) Greet and frame	Mrs. X, thank you for coming to this interview. The purpose of this interview is to talk about the depression prevention program that you have started on your mobile, and we will be here for approximately 15 minutes
2) Open-ended initial question	How did the mobile-based depression prevention plan go?
3) Active listening about his/her risk and protective factors for depression	What did you understand from the report that was given to you on your mobile phone about your responses to the questionnaires? The report indicates that you have problems with…
4) Detect emotions and empathize	I see that you are having a hard time; it really is a difficult situation...
5) Invite the patient to verbalize attitudes and behaviors that they are already using to prevent depression	As you already know, at this time, you are not depressed and this is due in part to the fact that you are already doing things to prevent depression, could you share any of them with me?
6) Reinforce those attitudes and behaviors that have scientific evidence in favor of the prevention of depression and discourage those that are risk factors for depression	Exercising does indeed prevent depression...; however, drinking three whiskeys before going to sleep is not a good way to prevent depression and may even be a risk factor that contributes to poor sleep and the onset of a depressive episode
7) Invite the patient to suggest new attitudes and behaviors to prevent depression	In addition to the things you have told me that you already do to prevent depression, is there anything else you could also do to prevent it?
8) Ask about the personalized prevention plan (PPP) that the app has suggested and help them understand it	What do you think of the PPPs that have been suggested on your mobile phone? Which of the prevention programs would you like to try? Is there any prevention program that you are unclear about?...
9) Strengthen adherence to the intervention	Your participation in this program will possibly improve your physical and mental quality of life and will also help you to deal more effectively with your problems, so I encourage you to carry out the tasks of the prevention programs that you choose on your mobile
10) Closing the interview and communicating your “open door” attitude	Well, Mrs. X, this interview has concluded. We won't have any more scheduled interviews to talk about this program. However, if you have any questions or want to ask me something about this depression prevention program, you can do so whenever you wish during the year of study follow-up

#### The e-predictD intervention modules

The e-predictD intervention has a biopsychosocial orientation and is based on scientific evidence. The e-predictD app includes eight intervention modules, six psychological and/or psychoeducational modules based on principles of cognitive behavioral therapy (improving sleep, problem-solving, communication skills, decision-making, assertiveness, and working with thoughts), one physical-based intervention (physical exercise), and a social-based intervention (improving relationships).

All e-predictD intervention modules are self-guided, have a similar structure, and several are interrelated. They have (1) an initial part on definitions and educational and motivational messages; (2) an initial evaluation of each specific area of the intervention module; (3) a pool of recommendations; (4) exercises to apply/practice what has been learned; (5) reminders for working on what has been learned; (6) a set of support tools (videos, bibliography, and links to other intervention modules); and (7) a review of compliance with the objectives and commitments defined by the users themselves. Some of the specific characteristics of each intervention module are described below:

- The “physical exercise” intervention module aims to reduce sedentary lifestyles in those patients previously identified as sedentary. There is evidence that exercise-based interventions prevent depression and the effect size of psychological and exercise-based interventions could be similar (40). Therefore, if patients have these two alternatives, the impact on the prevention of depression would be even greater, since those patients who are poorly motivated by psychological programs could be more motivated by physical exercise programs and vice versa. Depending on the contexts (age, physical limitations, own resources, etc.) and preferences, patients can select the type of exercise that most motivate them to practice (gymnastics, walking, cycling, dancing, team sports, etc.). In addition, this module offers a set of videos for performing exercises at home. Finally, this module also offers a list of community resources to practice physical exercise in the neighborhood.
- The “social relationships” intervention module aims to increase interpersonal relationships in those patients who perceive low social support. A few weeks before the start of the e-predictD intervention, the social workers of each PCC collect and update all the community resources available, which include group activities in the neighborhood where the PCC is located and the patient therefore resides. This list and its basic information (name, telephone, web and physical address, activity of interest related to the prevention of depression, and free or payment required) are entered in this intervention module. Patients should choose any of these available community resources and participate in their activities over time. Moreover, patients and GPs could discuss it in the patient–GP interview to help them decide. Social prescription intervention through community resources could improve perceived social support and thus contribute to the prevention of depression (62). When it is identified that a patient has an interest in improving a relationship with another person, in particular, the patient is redirected to the communication skills module and/or problem-solving module when this relationship is perceived as a problem.
- The “problem-solving” intervention module aims to help people to find solutions to any problem they want to resolve. There is some evidence of the effectiveness of “problem-solving” interventions in the prevention of depression (63, 64). Dysfunctional attitudes, worrying, a negative problem orientation, and perceived control all play a mediating role in cognitive behavioral therapy (CBT) as well as in problem-solving therapy (65). This module provides instruction on how to apply problem-solving strategies. First, the module helps people to define, concretize, and properly communicate the problem. Then, it deals with turning the problem into an objective to negotiate it and reach agreement. People have to recognize what is important to them and what is expendable and can be offered in negotiation. Exercises to apply/practice these strategies are provided, and then, the module requests feedback on practicing those strategies. In addition, this module offers the possibility of connecting with the communication skills, assertiveness, and decision-making modules whenever the person deems it necessary.
- The “communication skills” intervention module aims to provide instruction on communications skills to people who identify difficulties communicating with others. Patients obtain information regarding the principles of good communication, considering active listening skills and both verbal and non-verbal communication. In addition, they are encouraged to practice communication skills with others in real situations. Frequently, the module provides reminders to work on these skills and requests feedback on practicing those skills. This intervention module is based on some of the techniques used in both CBT and interpersonal therapy.
- The “assertiveness” intervention module aims to improve communication skills in difficult communicational situations (such as how to say no, make a request, or defend your own ideas and opinions) in people who feel very uncomfortable dealing with these situations. This intervention module provides instruction on identifying assertive sentences and offers situations with different response options and feedback according to each response. It also includes exercises for practicing with difficult communicational situations adapted to the needs of patients, as well as reminders to work on the skill and to provide feedback on the task performed. This intervention module is related to the previous one, but it is focused on this specific communication skill. Working on assertiveness is another technique included in the programs to improve interpersonal relationships offered by CBT and interpersonal therapy.
- The “decision-making” intervention module aims to help people who have to make these decisions by reflecting on them and considering the advantages and disadvantages of the different options. This module provides instruction on applying the steps of decision-making strategies. First, people are instructed to define and concretize the decision that they want to make. Second, they are trained to identify several alternatives regarding a decision. Then, patients are encouraged to work on their own decisions, reflecting on and analyzing the advantages and disadvantages of the different alternatives. Finally, patients commit to practicing these strategies in a real setting and this intervention module requests feedback on that practice. These strategies are also included in effective CBT for the prevention of depression.
- The “improving sleep” intervention module is directed to patients who perceive relevant sleep problems. Many people who suffer from depression have insomnia, and insomnia in turn is a risk factor for depression (66). There is also evidence that interventions aimed at improving sleep prevent depression (67). This intervention module provides information about good sleep habits (sleep hygiene recommendations) and encourages people to follow those recommendations. Every week, the program requests compliance with the recommendations. In addition, the module suggests keeping a diary of sleep quality.
- The “working with thoughts” intervention module aims to reduce automatic negative thoughts and worries. Cognitive change is the most evaluated and involved mediator in the prevention of depression and anxiety (42). This intervention module provides instruction on how to identify and analyze automatic negative thoughts and turn them into positive thoughts. The sequence is as follows: first, learn to understand cognitive bias; second, identify cognitive bias; and third, change any cognitive bias discovered (negative thoughts). Later, a series of activities are carried out to generate positive emotions that replace negative thoughts. Concerning worries, the goal is to analyze and reduce them. Time is allotted for people to learn to identify worrying. Once this has been learned, people analyze their worries ranking them by relevance and priority and reduce them through relaxation exercises, mindfulness, psychoeducation, and monitoring. Patients can prioritize work with certain thoughts and dispense with others (e.g., worry vs. negative thoughts).

#### The e-predictD monitoring system

**Figure 8:**
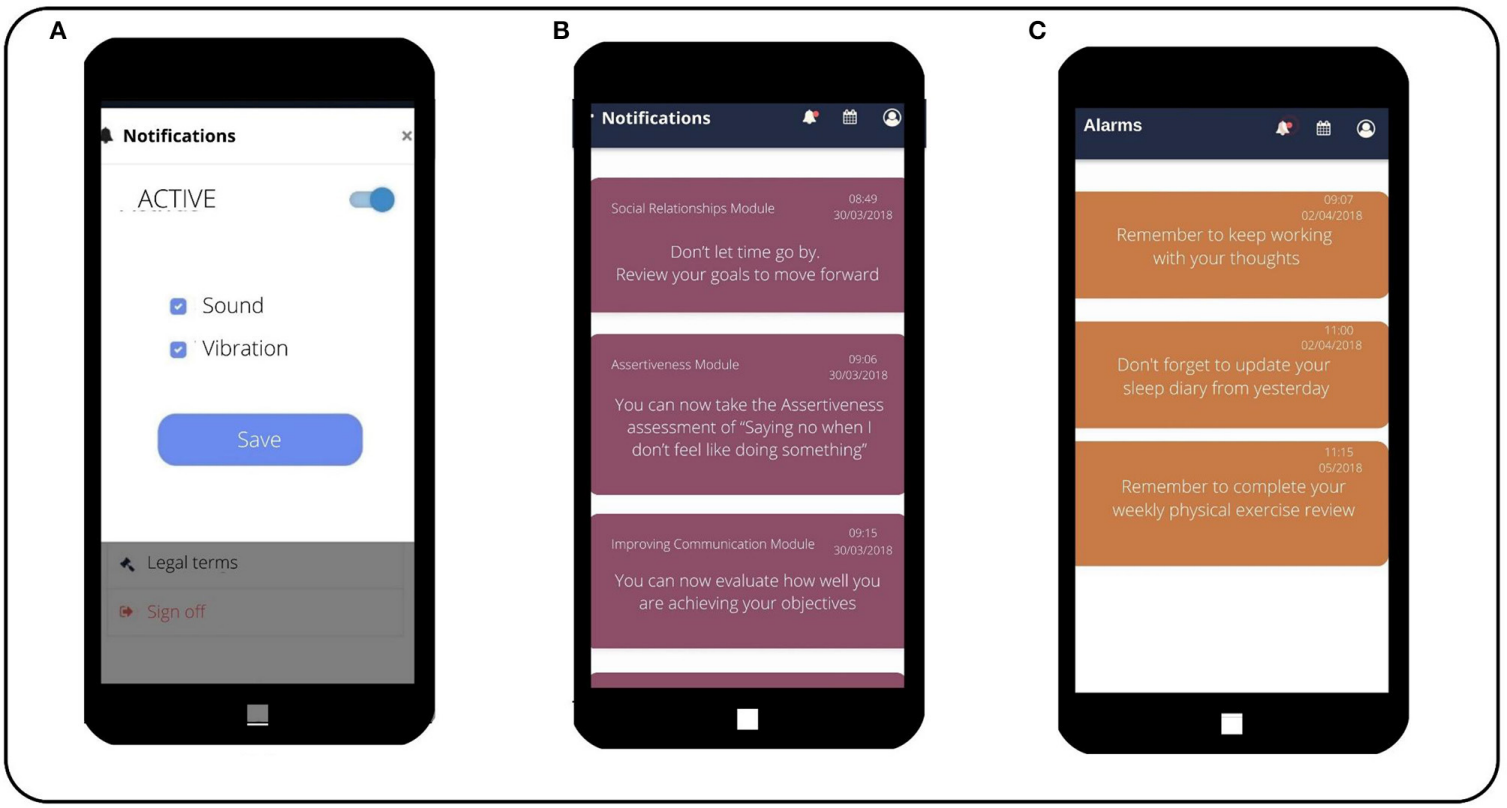
The e-predictD monitoring system. **(Screen A)** System regulation Notifications and monitoring by the app. **(Screen B)** Notifications for the patient regarding the content of the different modules active in the app. **(Screen C)** Alarms and reminders for the patient regarding evaluations or entries for the different modules that are active.

Once patients choose the PPP to be self-implemented in the next 3 months, a monitoring and support plan for each specific PPP is established. The DSS will add new decision algorithms based on the comparative information of the previous assessments. For example, if the risk level for depression has increased at 3 months from moderate to high and the DSS and the patient did not previously select the ‘working with thoughts’ intervention module of the PPP, this will be suggested in his/her new PPP. This decision is based on the available evidence on the hierarchy of mediators involved in the prevention of depression (42). The e-predictD monitoring system will send messages, brief reports, tracking charts, and alerts throughout the study (see Figure 8). If patients consent, their GPs will also have access to this monitoring information and will be able to check their patient's e-predictD report, completed assessments, and the modules in which their patients are working (PPP) at any time.

### The intervention for the control group

**Figure 9:**
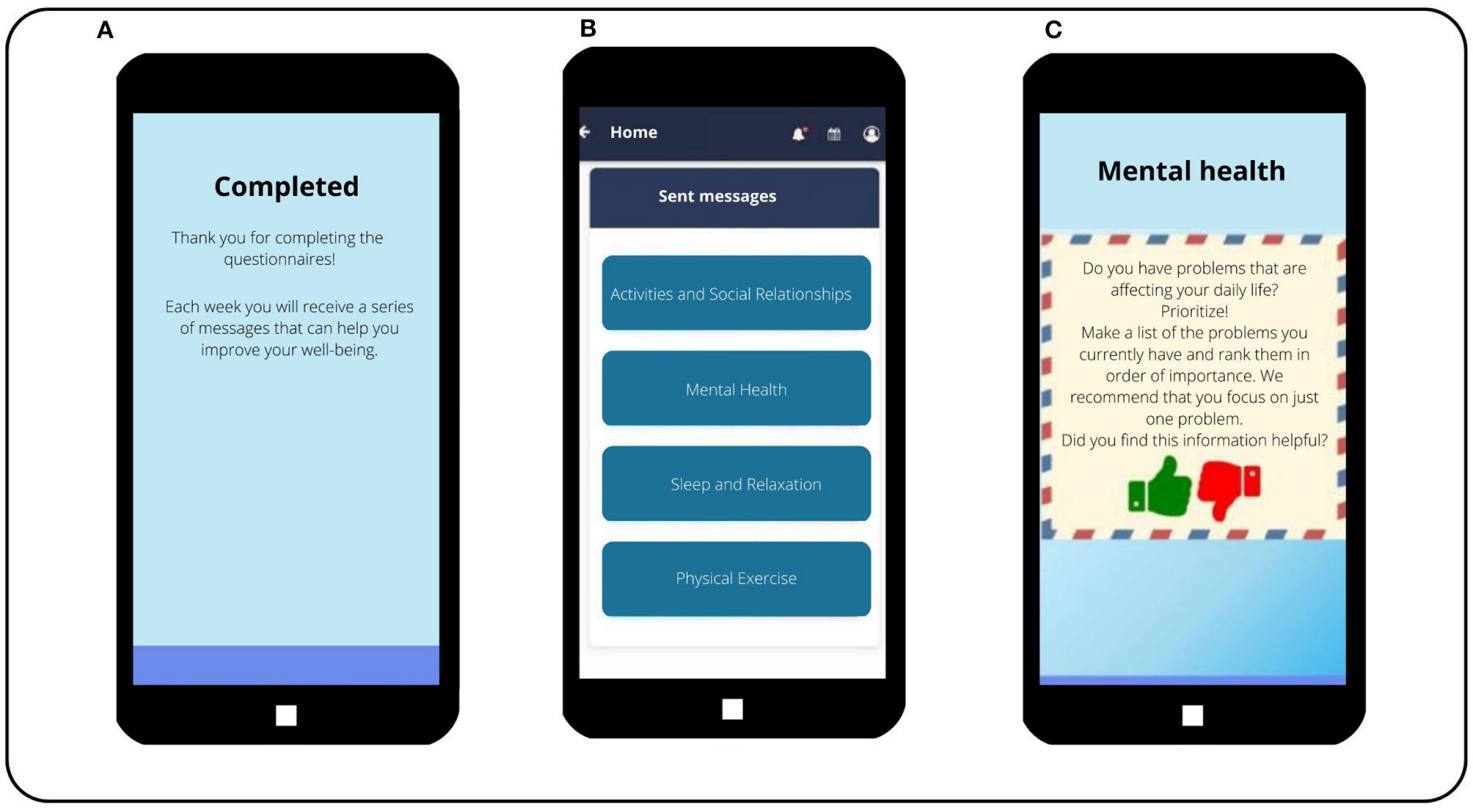
The e-predictD intervention for patients in the active-control group. **(Screen A)** Once a patient in the control group has completed the questionnaires, he/she does not receive a report. Instead, this informational message is sent to the patient. **(Screen B)** In the main menu of the control group app where the boxes with the messages they will receive are displayed, a total of 52 messages will be sent each week for the next 12 months. The messages are stored in these four blocks. **(Screen C)** Message sent to a patient in the control group. It also contains an evaluation question that the patient must answer.

Patients of both arms will continue to receive CAU from their health providers. Patients assigned to the control group download an alternative version of the e-predictD app that looks very similar to the intervention group's version. They will receive brief messages about general health information and mental health in particular (active control group). This information will be sent weekly for 52 weeks through the e-predictD app and will be extracted from brochures, clinical practice guidelines, and official websites (e.g., WHO; see Figure 9). However, in the active control group, there will be no personalization, information on the level of risk, risk factors, or internal–external resources to prevent depression. Nor will there be interaction, PPPs, or an initial GP–patient interview. GPs in the control group will not receive any training. Patients of both intervention and active control groups will receive the same notification and reminders to complete the questionnaires during follow-up.

### Safety protocol

In any trial concerned with mental health, there is the potential for psychological distress in participants. Patients in either arm falling into PHQ-9 or/and Generalized Anxiety Disorder (GAD-7) score above 14 and/or reporting suicidal ideation (“nearly every day” in item 9 of the PHQ-9) at baseline and/or during follow-up will be automatically informed regarding their mental health. Through sequential messages over time, they will be suggested to seek further help from their GPs. Additionally, an optional call-back service for individuals requiring further support or direction will be provided. This call-back will occur within 3 days by a staff psychologist who will guide participants into necessary care arrangements.

### Outcomes, variables, measurements, and follow-up

For an overview of instruments used at baseline and at 3-, 6-, 9-, and 12-month follow-up assessments, see Table 1.

#### Outcomes

Our primary outcome is the cumulative incidence of major depression during 12 months of follow-up, assessed through the depression section of the CIDI (68) at 6 and 12 months. As secondary outcomes, we will use measurements of mental and physical quality of life using the SF-12 (69), depressive and anxious symptoms [PHQ-9 (70) and GAD-7 (71)], and the probability of the onset of major depression at 12 months (predictD risk algorithm) (28). All of these will be evaluated at baseline and at 3, 6, 9, and 12 months. We will also make estimates of cost-effectiveness and cost-utility (see economic evaluation below).

#### Variables and measurements

The questionnaires and questions were self-completed online by patients at baseline and at 3, 6, 9, and 12 months; except for the assessment of PHQ-9 and the predictD risk algorithm at baseline. These were collected face-to-face by the research assistants in the screening and these data were automatically incorporated into the database through a web application, along with the rest of the self-completed measures at baseline.

- The Patient Health Questionnaire (PHQ-9) (70) includes nine items that evaluate the presence of depressive symptoms in the last 2 weeks. Each item has response options ranging from 0 to 3 (0 = “not at all,” 1 = “several days,” 2 = “more than half of the days” and 3 = “nearly every day”). The score range is 0–27. The PHQ-9 showed good psychometric properties in Spain (53).
- The Generalized Anxiety Disorder questionnaire (GAD-7) (71) includes seven items measuring anxious symptoms in the last 2 weeks, which are based on the DSM diagnostic criteria for GAD. The GAD-7 is also an effective screener for panic, social anxiety, and post-traumatic stress disorders (72). Response options are similar to those of the PHQ-9, and the score ranges from 0 to 21. The Spanish version of the GAD-7 also had good psychometric properties (73).
- The Spanish predictD risk algorithm calculates the individual probability of the onset of major depression in the next 12 months. The predictD-Spain algorithm has been internally validated in Spain and externally validated in Chile and other European countries, showing excellent calibration and discriminant validity (C-index 0.82) (28). The predictD-Spain risk algorithm outperformed the predictD-Europe risk algorithm by 5.6 Integrated Discrimination Improvement (IDI) points when used in Spanish primary care attendees (28). The predictD-Spain algorithm comprises a set of 11 predictors:

1. Spanish province.
2. Age.
3. Gender.
4. Educational level (beyond secondary education, secondary education, primary education, or incomplete primary education/illiterate).
5. Quality of life, using the SF-12 (69). This questionnaire consists of two components, one related to physical health and another related to mental health. The scores range from 0 to 100; higher scores indicate better health-related quality of life. The SF-12 was validated in the Spanish population and demonstrated adequate reliability and validity (74).
6. Controls, demands, and rewards for unpaid work using an adapted seven-item version of the Job Content Instrument (75). This questionnaire consists of questions about difficulties in unpaid work, the frequency with which help is perceived, and satisfaction with this unpaid work. The result is classified into three categories (satisfied, dissatisfied, and very dissatisfied) through the sum of the seven items. This questionnaire showed good psychometric properties in Spain (76).
7. Satisfaction with living together at home, using an option from the five-item Likert response scale ranging from 1 (very dissatisfied) to 5 (very satisfied).
8. Presence of serious problems in relatives or close persons, using four different dichotomous response (yes/no) items on serious physical, psychological, and substance misuse problems and/or disability.
9. Childhood experiences of physical abuse, using an option from the five-item Likert response scale ranging from 1 (never) to 5 (frequently).
10. Screening for lifetime depression, using the first two questions included in the CIDI about lifetime low mood (77).
11. Medication consumption for anxiety, depression, or stress, in the previous 6 months through an item with a dichotomous answer (yes/no).

- Two questions from the Spanish version of the Duke-UNC-11 instrument for evaluating perceived functional social support, one referring to affective social support and another to confidential social support subscales. Both items have the highest item-subscale correlation. Each item has response options ranging from 1 (“much less than I wish”) to 5 (“as much as I wish”) (78).
- Physical activity using a brief physical activity assessment (79, 80) consisting of two questions, one that assesses the frequency and duration of vigorous-intensity physical activity and another that assesses the frequency and duration of moderate-intensity physical activities (including walking) undertaken in a week. A score >4 = “sufficiently” and 0–3 = “insufficiently” active.
- A question about whether the participants have difficulty saying ‘no’ to other people with four Likert response options ranging from 0 (never) to 5 (frequently).
- A question about whether the participants have difficulty communicating with other people with four Likert response options ranging from 0 (never) to 5 (frequently).
- A question about whether the participants have a conflict or problem with someone and they want to resolve it (yes/no).
- A question about whether the participants have difficulties making decisions (yes/no).

The questionnaires hetero-administered by telephone at 6 and 12 months are the following:

- Depression section of the CIDI. The CIDI is a structured psychiatric interview that was designed and evaluated by the WHO (68), which has excellent reliability and validity in different cultures and populations (81, 82). Through the CIDI, we will obtain diagnoses of major depression based on DSM criteria.
- To assess the attitudes and satisfaction of the patients regarding the e-predictD and the active control interventions, we use an adaptation of the e-Health Impact Questionnaire (e-HIQ) (83) selecting 17 of the 37 items included in the e-HIQ. This is hetero-administered at 6 and 12 months of follow-up. In addition, we have added an open-ended question to assess the appearance of adverse effects attributable to the e-predictD intervention or to the active control group: “In the last 6 months, have you noticed any discomfort related to the depression prevention intervention you are receiving? If so, please describe it in your own words.”

The questionnaires hetero-administered by telephone at baseline and at 6 and 12 months are the following:

- Healthcare resource use will be evaluated using the Client Service Receipt Inventory (56), which collects information on the use of services, psychotropic drugs, sick leave, and loss of productivity.
- Quality-adjusted life years (QALYs) are measured using the EuroQol five dimension questionnaire (EQ-5D-3L) (84, 85). This is a widely used measure of general health and quality of life, which includes five domains that address mobility, self-care, usual activities, pain/discomfort, and anxiety/depression, and three answer options (no problems, some problems, and extreme problems). In addition, it includes a visual analog scale graded from 0 (the worst health status) to 100 (the best health status). Spanish tariffs will be used to estimate the utility of health states described by the patients (86).

The questionnaires and questions self-completed at baseline by the GP participants in both arms of the trial are the following:

- Spanish Province.
- Primary care center (PCC)
- Age.
- Gender.
- Job characteristics: urban or rural PCC, type of contract (permanent or temporary/substitute), months working at the current PCC, patient list size, and mean time per visit.
- Training: year of medical degree earned, postgraduate training (3 or 4 years) in general and family medicine (yes/no), accreditation as a GP tutor (yes/no), experience in training GPs [first-year resident assigned (yes/no), and/or third- or fourth-year resident assigned (yes/no)], and membership in a Spanish health and communication group (yes/no).
- Satisfaction with the management of patients with mental disorders in primary care: satisfaction with the communication and care shared with the mental health team (secondary care; five Likert response options), satisfaction with the role of the primary care nurse in mental health disorders (five Likert response options) and satisfaction with the role of the primary care social worker in psychiatric disorders (five Likert response options or “I do not have a social worker in my health center”).
- Profile of professional practice: A three-dimensional questionnaire validated in Spain, with four items each and response options from 1 = “strongly agree” to 4 = “strongly disagree” concerning professional satisfaction, workload perception, and biomedical vs. psychosocial orientation (87).
- Clinicians' attitudes toward depression will be evaluated using the Revised Depression Attitude Questionnaire. This questionnaire consists of 22 items on professional confidence in depression care, therapeutic optimism, and views on generalist or specialist perspectives pertinent to depression and its care (88, 89). The response options range from 1 (strongly disagree) to 5 (strongly agree) using a 5-point Likert scale.

#### Adherence

To improve study adherence, a research assistant will call participants when the following situations occur: (1) participants do not attend the interview with their GPs, (2) participants have not used the app (which means they have not entered any intervention module) after the visit with their GPs, and (3) participants have not self-completed any follow-up questionnaires.

### Data analysis

#### Power and sample size calculation

To calculate the necessary sample size, we estimate that in the control group, there will be an incidence of major depression of 20% in the year of follow-up since we have previously screened the patients at moderate-to-high risk for depression. We also expect that in the intervention group, the incidence of depression will be 11.2% (absolute risk difference = 8.8%; relative risk = 0.56). In the e-predictD study, we expect a greater effect size than in the predictD study because the latter implements a very simple intervention (a 15-min patient–GP interview every 6 months) for the universal prevention of depression; while in the former, the intervention is more intense and personalized and its target population is at a moderate-to-high risk. We assume an alpha error of 5% and a power of 80% for a two-sample Pearson's chi-squared (two-tailed proportion test) and cluster randomized design: 72 GPs (36 GPs for each group) and 10 patients per GP, assuming that the distribution of the cluster will be homogeneous (CV <0.23) (90), and an intracluster correlation coefficient for GPs of 0.04, which was obtained from a previous study (29). Therefore, we would need to recruit 720 patients in total, 360 for each arm of the trial.

#### Statistical analyses

We will perform all statistical analyses based on the intention-to-treat principle, analyzing all participants according to their randomized treatment and including all of them in the analyses. Although the allocation is by cluster (GPs), inferences will be made exclusively at the patient level. All the analyses will be performed with Stata, version 17 (StataCorp). We will use multiple imputations for dealing with missing data under a missing-at-random framework. The multiple imputations by chained equations procedure will be conducted to obtain at least 20 imputed datasets, and Rubin's rules will be used to combine them (91). To analyze the effect of the intervention on each outcome measure, we will use hierarchical logistic or linear regression models for clustered data. Our primary analysis will be the absolute risk difference in the cumulative incidence of major depression at 12 months of follow-up, adjusted *a priori* for risk of depression at baseline because it is considered strongly predictive of the outcome and thus clinically prognostic (92). Our secondary analyses will include as dependent variables the respective scores for depressive (70) and anxious (71) symptoms, the probability of the onset of major depression in the next 12 months (29), and mental and physical quality of life (69). We will conduct multilevel linear regressions including the GP and time variables as random components, and the variables group, time, the interaction group^*^time, and the respective measurements of the dependent variables at baseline as fixed components. We will calculate standardized mean differences using the margins in Stata. Sensitivity analyses will be conducted: (1) adjusting for those variables unbalanced at baseline between the arms of the trial; (2) performing complete case analysis and adjusting for inverse probability weighting (93) to minimize attrition bias during follow-up. We will evaluate some moderators (age, sex, educational level, anxiety and physical comorbidity, first lifetime depressive episode, and level of risk for depression) and mediators using multilevel structural equation models to describe the causal chain as Kraemer suggests (94). Finally, we will use different definitions of compliance with adherence to the intervention and perform a complier average causal effect analysis (95).

#### Economic evaluation

The economic evaluation will be conducted from two perspectives: (1) societal, including the costs of all types of health services (direct costs) and the costs that stem from production losses (indirect costs), and (2) National Health System (including only direct costs from Spanish public health services). As stated above, costs will be estimated through a modified version of the Client Service Receipt Inventory (56). Direct health costs will be calculated by multiplying the number of health service units (consultations, hospital days, tests, etc.) by their standard price and the cost of the medication used by multiplying the cost per daily dose (obtained from the Spanish Pharmaceutical Vademecum, http://www.vademecum.es/) by the number of prescription days recommended. Indirect costs consist of the costs of absenteeism from paid work. Costs of work loss will be calculated by multiplying the days on sick leave by the minimum daily wage in Spain according to the human capital approach. In addition, self-reported presenteeism will be assessed using some questions from the World Health Organization Health and Work Performance Questionnaire (96).

The costs of GP training and visits attributable to interventions in both arms will be added. The QALYs will be calculated using the EuroQol-5D-3L (84). Official Spanish tariffs will be used to estimate the utility of health states described by the patients (86) and linear interpolation will be used for transitions between health states.

To calculate incremental cost-effectiveness ratios, incremental costs (cost difference between the intervention and comparator condition) will be divided by the incremental health effects (the effect differences between conditions). To calculate the ‘incremental cost-utility ratio’, the incremental effect is the differences in QALYs between conditions. The incremental costs, health effects, and QALYs will be modeled by generalized linear models to account for clustering effects (GP as a random component) and explore the family and correct link of the models (97). All models will be adjusted by their respective baseline values (i.e., QALYs or cost), the individual risk of depression (predictD risk algorithm), and those variables unbalanced at baseline if necessary. The confidence intervals and the plans and cost-effectiveness acceptability curves will be generated with bootstrapping for each imputed data file (97), varying the values of the availability to be paid from € 0 to € 100,000. Sensitivity analyses will be carried out by modifying unit prices and statistical analyses (e.g., using seemingly unrelated regressions), using the average salary instead of the minimum salary and including the loss of productivity.

## Discussion

To the best of our knowledge, the e-predictD study will be the first to evaluate the effectiveness and cost-effectiveness of a personalized biopsychosocial intervention based on ICTs, risk prediction algorithms, and DSS for the prevention of depression in primary care. We propose an innovative intervention to prevent depression in primary care. The e-predictD intervention has a broader focus, the biopsychosocial orientation, which goes beyond the exclusively psychological approach. The e-predictD intervention also incorporates elements of “P4 medicine” (precision, predictive, personalized, and participatory medicine) (42), involving GPs and patients and using technological solutions based on ICTs, risk prediction algorithms, DSS, and monitoring and feedback systems. The e-predictD study incorporates approaches based on the UK Medical Research Council for complex interventions, involving different stakeholders in the design and evaluation of the e-predictD intervention, using mixed methods research (qualitative and quantitative), a previous pilot study, and a multicenter cluster randomized trial to evaluate the effectiveness and cost-effectiveness of the e-predictD intervention in preventing depression. The e-predict study also presents several limitations:

(1) The GPs who choose to participate in the e-predictD study may have a different profile (psychosocial orientation, different training or work satisfaction, etc.) to those who do not. Similar biases could occur if a large proportion of patients refuse to participate. It is not easy to obtain information from those GPs and patients who refuse to participate, so it will be difficult to ascertain the direction and magnitude of this possible selection bias. (2) This potential selection bias could also limit external validity (98). In addition, the inferences of this trial will be limited to primary care attenders aged 18–55 years at moderate-to-high risk of depression with internet access and basic technological literacy. Nonetheless, primary care is an ideal setting to carry out primary prevention strategies for depression because many people at risk of depression go to see their GPs. (3) Apart from the only per protocol visit that patients will have with their GPs, the e-predictD is basically an intervention that is unguided and delivered via smartphone; therefore, trial attrition and disengagement are potential issues (99). Reasons for attrition are complex and, in reality, there are contradictory possibilities of drop-out due to dissatisfaction or lack of engagement, as opposed to drop-out due to a sense that the individual feels his/her needs have been met. We will use those evidence-based strategies to maximize engagement and adherence: reducing the length of follow-up questionnaires and giving participants feedback on their progress while filling out the questionnaire, automated tailoring, real-time engagement, gamification and intrinsic motivation to engage, log of past app use, reminders to engage, and a simple intuitive interface and interactions (100). To address the possibility that users may delete the app during the trial, which may hamper follow-up attempts, later follow-ups will occur via the phone numbers provided, rather than via the app. Participants will receive up to three phone calls to complete each assessment and follow-up measures will be accepted as long as they are obtained within 90 days of the planned assessment. In addition, we will use a robust statistical method (multiple imputations) for data missing at random, so we hope that this limitation will be minimized. (4) Although cluster randomization by GP will reduce possible contamination bias, some degree of contamination could occur due to the coexistence in the same PCC of GPs and patients of both control and intervention arms. The magnitude of this bias would be small, but in any case, it would go against the effectiveness hypotheses of the e-predictD study. (5) Since we evaluate a psychosocial intervention in real practice, it is not possible to blind GPs who provide it (55). Both the intervention and active control GP groups could modify their behavior because they feel observed (Hawthorne bias). These behavioral changes in the GPs in the active control group could be influenced by social desirability bias, whereby they could improve the usual care they provide to their patients in the control group; therefore, this last bias would also go against the hypothesis of this study. (6) We use an active control group, whose intervention (brief weekly psychoeducational messages) could have some preventive effect on depression, especially in those patients with a lower risk of depression, which could reduce the differences in the preventive effect between both the e-predictD and the active control groups. (7) The cluster randomization might produce a good balance between the groups at the GP level; however, this does not guarantee a good balance at the patient level (101), which could contribute to confounding bias. We will perform adjusted analyses for variables unbalanced at baseline if necessary. (8) Finally, there could be post-randomization biases (e.g., unbalanced use of antidepressants between the intervention and active control groups during follow-up), which will be addressed by carrying out measurements and adjusted analyses.

If the e-predictD intervention proves to be effective and cost-effective in preventing depression and also has good acceptability and adherence by patients and GPs, its overall impact could be substantial, reducing overall depression burden as well as associated healthcare cost and improving mental health and quality of life. This overall impact would be relevant even if the size of the preventive effect was relatively small since its implementation in primary care and through apps would facilitate its scalability to a large part of the population at risk of depression.

## Ethics statement

This protocol involving human participants was reviewed and approved by the Ethics Committee in each participating Spanish city: Regional Research Ethics Committee of Malaga, IDIAP Jordi Gol Clinical Research Ethics Committee (Barcelona), Clinical Research Ethics Committee of Aragon (CEICA), Clinical Research Ethics Committee of the Health Area of Salamanca. The participants provided written informed consent to participate in this study.

## Data management

The management of the study data will be carried out in accordance with Regulation (EU) 2016/679 of the European Parliament and of the Council of April 27 2016 on the protection of natural persons with regard to the processing of personal data and the free circulation of these data; and also in accordance with Spanish laws: Organic Law 3/2018, of December 5, on the Protection of Personal Data and guarantee of digital rights, as well as Law 41/2002, of November 14, basic regulating autonomy of the patient and of rights and obligations regarding information and clinical documentation, among others. All study participants will be informed about the purpose of the study and the use and reuse of the data. They will sign an informed consent to join the study, which, together with the study protocol, has been validated by the competent ethics committees. After recruitment, the personal data of the participants will be removed from all data and stored in a password-protected encrypted database. Each participant will receive a code that will only be linked to the data, except in case of medical emergency. To ensure data quality, the research coordinator or person designated by him/her will implement and maintain quality assurance and control procedures with written standard operating procedures to ensure that the study is conducted and that the data is generated, documented, and reported in accordance with the protocol. Quality control procedures will be implemented starting with the data entry system, and data quality control checks will be performed on the database. The data, which will be collected in the framework of this project, will be used to study depression prevention. Access to the data will be through an administrator user in a back office or through export to CSV format. The data will belong to the research group according to the privacy policy and will be located in a database within servers, which will be blocked with encrypted files and passwords. The project research team will be responsible for the curation-purification and conservation of the data. For the preservation of the data, periodic backup copies will be made.

## Data sharing

The data generated during this study will include detailed information on the protocols and analyses used, to allow their reproducibility and future analyses by other groups. We recognize and support the principles of data sharing. The data generated from this research will be made available to affiliated investigators through secure and anonymized databases. Only investigators with specific independent ethics committee approval will have access to any anonymized data. The research coordinator of the e-predictD study can be contacted for de-identified data requests. Consent for such data sharing will be integral to enrollment in the study, and our participants will be asked about their willingness to have their data shared to advance health research.

## Author contributions

JAB, AR-M, and PM-P led the scientific specification, conceptualization of the study protocol, and drafted the manuscript. SC-C, HC-P, AR-B, MIB-R, ER-S, JMM, YLH, JDL, and OT-M contributed the scientific specification, conceptualization of the study protocol, and conducted a critical revision of the manuscript for important intellectual content, provided feedback, discussed, and approved the final manuscript. All authors contributed to the article and approved the submitted version.
